# The Role of Financial Incentives Along the Antiretroviral Therapy Adherence Continuum: A Qualitative Sub-study of the HPTN 065 (TLC-Plus) Study

**DOI:** 10.1007/s10461-017-1821-7

**Published:** 2017-06-13

**Authors:** Elizabeth E. Tolley, Jamilah Taylor, Allison Pack, Elizabeth Greene, Jill Stanton, Victoria Shelus, Richard Dunner, Theo Hodge, Bernard Branson, Wafaa M. El-Sadr, Theresa Gamble

**Affiliations:** 1 Behavioral, Epidemiological & Clinical Sciences Division, FHI 360, 359 Blackwell Street, Suite 200, Durham, NC 27701 USA; 2 Health Behavior Department, Gillings School of Public Health, University of North Carolina, Chapel Hill, NC USA; 3 Science Facilitation, FHI 360, 359 Blackwell St, Durham, NC USA; 4 Institute for Reproductive Health, Georgetown University, Washington, DC USA; 5 Narco Freedom, Bronx, NY USA; 6 Capital Medical, Washington, DC USA; 7 Scientific Affairs LLC, Atlanta, GA USA; 8 ICAP at Columbia University, New York, NY USA

**Keywords:** Adherence, Stage of change, Financial incentives, HIV, United States

## Abstract

The stages of change (SOC) theory suggests individuals adapt incrementally to behaviors like adherence, requiring different strategies over the behavior change continuum. Offering financial incentives (FIs) is one strategy to motivate adherence. This qualitative sub-study examined adherence barriers and the role of FIs to increase viral suppression (VS) among HIV Prevention Trials Network (HPTN) 065 study participants categorized into SOC-related adherence stages based on changes from baseline to follow-up viral load tests. Of 73 participants, most were in Maintenance stage (n = 31), defined as having achieved VS throughout HPTN 065, or in Action stage (n = 29), defined as moving from virally unsuppressed to suppressed in 50% or more of tests. Only 13 were Low Adherers, having achieved VS in fewer than 50% of tests. The latter group faced substantial social and structural adherence barriers. Participants in the Action stage made positive changes to adherence routines to achieve VS. Those in Maintenance were less incentivized by FIs, as they were already committed. Results from this sub-study suggest FI effectiveness may vary across the SOC continuum, with greatest impact for those initiating antiretroviral or without explicit adherence routines. FIs may be insufficient to overcome strong social or structural barriers, and unnecessary for those intrinsically committed to remaining adherent.

## Introduction

The HIV Prevention Trials Network (HPTN) 052 study demonstrated that antiretroviral (ART) therapy dramatically reduces the risk of transmission of HIV among sero-discordant couples [1]. This finding and the results of the START study, which demonstrated benefit from early ART initiation, motivated a revision of ART treatment guidelines to recommend ART for all HIV-infected individuals [2–4]. Improvements in medication regimens, reducing the number of pills and their toxicity, have facilitated patients’ adherence. Nevertheless, studies suggest that many patients do not achieve optimal adherence [5–8].

**Fig. 1:**
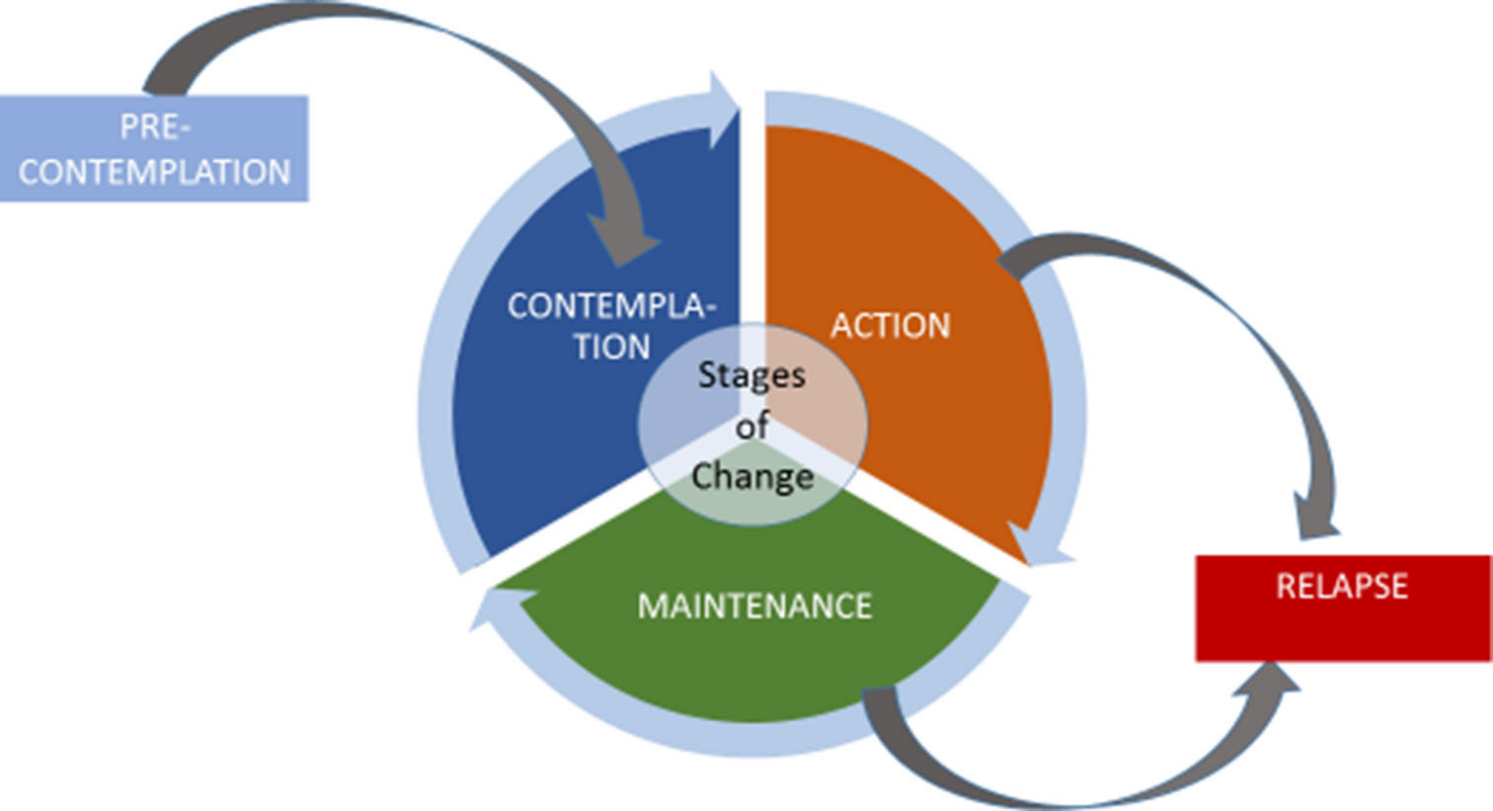
Stages of change

The stages of change (SOC) model (Fig. 1) has been applied previously to explain and/or predict ART medication adherence behaviors [9–13]. It posits that individuals adapt incrementally to new, complex behaviors like ART adherence by moving from early stages of contemplating and planning for change to testing out new behaviors and eventually maintaining the adopted behavior [14]. The model suggests that individuals may require or make use of different knowledge, skills and motivations as they move from one stage to another. Individuals in pre-contemplation, the first level of behavioral change, are either unaware of the need for change or do not intend to change their behavior in the near future, whereas those in contemplation and preparation phases are actively thinking about—and then planning for change. To move from pre-contemplation to contemplation or preparation, knowledge about one’s risk and the benefits of a therapeutic or preventive behavior is often a necessary condition, but plays little role in supporting the same individual to maintain a behavior once enacted. The SOC also suggests that anywhere along the continuum of behavior change people may relapse to earlier stages (Fig. 1).

Motivation has been identified as a critical element influencing an individual’s ability to initiate and sustain behavior change [15]. Whereas intrinsic motivation—undertaking a behavior to improve oneself—has been associated with longer-term behavior change [16], external sources of motivation, including the use of financial incentives (FIs), has been shown to motivate positive changes in a range of behaviors [17, 18]. Several studies have assessed the efficacy of FI to increase ART adherence, with varying results. For example, HIV-infected patients who experienced a previous treatment failure and received an adherence case management intervention with a $20 incentive at each follow-up visit were significantly more likely to show declines in viral load (VL) and improvements in immune function compared to those receiving standard treatment [19]. In contrast, no difference in viral suppression (VS) was found among HIV-infected drug users in Chennai, India who received FI [20] nor among hospitalized patients with HIV infection and substance use in the US [21]. Several small studies found initial improvements in ART pill-taking with provision of FIs, but adherence was not sustained [22–24].

The HPTN 065 (TLC-Plus) study, conducted between February 2011 and January 2013, evaluated the role of FIs in increasing VS through increased adherence to ART. Patients in care and on ART for a minimum of three months were eligible to receive a $70 gift card no more than once every 3 months if they were virally suppressed at their HIV care visits. The study found that the FI intervention increased VS overall with a stronger effect among participants who were not previously consistently suppressed [25].

Because no individual-level data were collected as part of the HPTN 065 study [26], a qualitative sub-study was designed to help interpret the results and explore patient and provider experiences with FIs. In this paper, we use changes in individuals’ baseline and follow-up VL test results to categorize their stage of medication adherence, examining whether and/or how the effect of FIs differed along the continuum of ART adherence stages. More specifically, we examine the role that FI played in helping participants achieve and/or maintain VS. The paper seeks to (1) identify what barriers and facilitators were experienced by patients receiving FIs in the HPTN 065 study, (2) examine whether these barriers and facilitators appeared to differ by a SOC-based adherence continuum, and (3) assess what role patients perceived the FI intervention to play in helping them adhere to their medication.

## Methods

We conducted semi-structured interviews with 76 participants from 14 clinic sites in Washington, DC and the Bronx, NY who participated in the HPTN 065 study. Sub-study participants were recruited by site staff after exiting the main study, following a non-probability, purposive, quota-based sampling strategy, to ensure that the qualitative sub-study included a heterogenous sample of patients in relation to their baseline VL status (suppressed or unsuppressed), ART initiation (before or after the study began) and exposure to FIs (≤3 or ≥5 gift cards). Standardized talking points were provided to each site for recruitment. Patients eligible for the sub-study must have been enrolled in care at the participating site, and eligible for the HPTN 065 study during at least 15 months of the 24-month intervention. VL test results obtained as part of the participants’ routine HIV care, beginning several months prior to study implementation and continuing throughout the study, and the date of ART initiation, were abstracted. VS was defined as HIV RNA <400 copies/mL. The sub-study was approved by the relevant Central or Local Institutional Review Boards (IRBs) prior to data collection.^1^ Written informed consent was obtained from all participants prior to data collection.

For this analysis, we categorized 73 of the 76 sub-study participants into 1 of 3 adherence-related categories, based on change between baseline VS and their VS status over the course of the study. Baseline VL test results were not available for three participants. The number of VL tests across sub-study participants was variable, as the intervention was added to standard HIV care in a clinical setting; thus, there was no study-specific visit schedule to adhere to, and patients were to continue with their normal pattern of HIV care visits. Participants who were not virally suppressed at baseline and on 50% or fewer of their subsequent VL tests were categorized as being in the Low Adherence stage. Participants were categorized as being in the Action stage if they had no VS at baseline, but achieved partial (more than 50% of VL tests with VS) or full VS (100% of VL tests) during the study. Those who were already on ART with VS at baseline and consistently maintained VS over the course of the study were classified as being in the Maintenance stage. Only 13 of 76 participants were ART naïve at baseline; we classified those who achieved more than 50% but less than 100% VS in the Action stage and those who were fully suppressed at each follow-up visit in the Maintenance stage (Table 1). In addition, we examined all 76 participants’ data for information about delaying ART initiation after diagnosis and/or periods off treatment to better understand pre-contemplation and relapse stages, as participants often described themselves in these stages prior to being in the study.

**Table 1:** Sub-study SOC groups and definitions

	Definition
SOC group during study	
Maintenance	ART-naïve at baseline and 100% VS during follow-up or on ART with VS at baseline and 100% VS over the course of the study
Action	ART-naïve at baseline and ≥50% but <100% VS over course of study; or on ART but not VS at baseline, but from 50 to 100% VS over the course of the study
Low Adherer^a^	ART-naïve or on ART but not VS at baseline and <50% of viral load tests suppressed over the course of the study
SOC categories experienced by all participants	
Pre-contemplation	Discussions about delaying ART treatment after HIV diagnosis from any sub-study participant
Relapse	Discussions about stopping ART treatment from any sub-study participant

^a^This group is similar to contemplation because they have not fully actualized adherence behavior

All interviews were conducted in English^2^ by trained interviewers after participants had exited the study. Interviews were audio-recorded and transcribed verbatim. Transcripts were then uploaded into NVivo 10.0 (QSR International) and analyzed thematically, following a process of reading, coding, data display and data reduction [27]. Members of the research team read a subset of transcripts to identify initial codes, both structural and emergent, for the dataset. Initial codes included: medication adherence, HIV testing, diagnosis and acquisition, opinions of the program, and impact on patient. Once the codebook was established, a team of analysts applied the codes in an iterative fashion, double-coding approximately 20% of patient interviews to assess intercoder reliability, discussing discrepancies, refining the codebook when interpretations differed, and recoding transcripts when necessary. Subsequently, two additional members of the research team read a subset of the transcripts to identify sub-themes related to medication adherence, which were then discussed and agreed upon by the sub-study research team. The two adherence team members then applied the second level of coding related to medication adherence to all transcripts, assessing intercoder reliability at several intervals. Sub-codes included adherence patterns, barriers to adherence, facilitators of adherence, medication attitudes and medication motivations.

For each participant group, we developed detailed memos that described self-perceived adherence patterns, barriers and facilitators to adherence, intrinsic and extrinsic motivations to adhere, attitudes towards FI and whether participants believed that receiving FI affected their adherence behaviors. Excel matrices included summary information from the memos (i.e., self-perceived adherence pattern reflecting high, low or mixed adherence) and incorporated information on the self-reported year of HIV diagnosis, HIV medication experiences, adherence barriers, facilitators, and socio-demographic information.

### Ethical Considerations

The qualitative sub-study was approved by the relevant Central or Local IRBs prior to data collection. Written informed consent was obtained from all interview participants prior to data collection.

## Results

The median age of participants was 48 (range 14–72) years. The majority were men (64%) and Black (58%). Approximately one-fourth of the sample were Hispanic (23%); half of participants self-identified as straight (49%), 40% as gay, and 10% as bisexual. Overall, participants’ characteristics generally resembled those of the larger sample of all participants exposed to the FI intervention [25]. However, some differences in socio-demographic and other characteristics exist across the three SOC adherence groups. In particular, the Low Adherence stage included a higher proportion of women and individuals diagnosed as infants or children than the other two groups (Table 2).

**Table 2:** Socio-demographic and behavioral characteristics

		All N = 73	Low Adherers N = 13	Action^a^ N = 29	Maintenance N = 31
Sociodemographic characteristics					
Mean age		44.1	37.8	44.7	45.3
Gender		% (n)	% (n)	% (n)	% (n)
Male		64 (47)	31 (4)	62 (18)	81 (25)
Female		33 (24)	62 (8)	34 (10)	19 (6)
Transgender (further information not collected)		3 (2)	7 (1)	4 (1)	0 (0)
Sexual identity					
Straight		49 (36)	86 (11)	51 (15)	32 (10)
Gay		40 (29)	7 (1)	34 (10)	58 (18)
Bi-sexual		10 (7)	7 (1)	0 (0)	6 (2)
Don’t know		1 (1)	0 (0)	15 (4)	3 (1)
Race					
Black		58 (42)	69 (9)	62 (18)	48 (15)
White		16 (12)	0 (0)	17 (5)	23 (7)
Not reported		26 (19)	31 (4)	21 (6)	29 (9)
Ethnicity					
Hispanic		23 (17)	23 (3)	21 (6)	26 (8)
Non-Hispanic		77 (56)	77 (10)	79 (23)	74 (23)
Education levels					
Some or no high school		31 (23)	54 (7)	38 (11)	16 (5)
High school/GED		25 (18)	23 (3)	21 (6)	29 (9)
Some college or associate degree		34 (25)	23 (3)	34 (10)	39 (12)
Bachelor’s degree or higher		10 (7)	0 (0)	7 (2)	16 (5)
Income					
$10,000 or less		52 (38)	77 (10)	59 (17)	35 (11)
>$10,000 </=$40,000		29 (21)	23 (3)	34 (10)	26 (8)
>$40,000 </=$80,000		12 (9)	0 (0)	4 (1)	26 (8)
>$80,000		5 (4)	0 (0)	0 (0)	13 (4)
Missing		1 (1)	0 (0)	4 (1)	0 (0)
ART naïve at baseline		18 (13)	0 (0)	14 (4)	29 (9)
% of follow-up VLs suppressed		85	37	89	100
Mean # of gift cards received		5.1	2.1	5.4	6.0
Behavioral characteristics (based on qualitative analysis)					
When diagnosed					
At least 20 years ago (1980s–1992)		22 (16)	23 (3)	17 (5)	26 (8)
Some time ago (1993–2006)		36 (26)	31 (4)	38 (11)	35 (11)
Recently (since 2007)		34 (25)	38 (5)	38 (11)	29 (9)
Unclear		8 (6)	8 (1)	7 (2)	10 (3)
Diagnosed as an infant/child		10 (7)	31 (4)	3 (1)	6 (2)
Pre-contemplation: described delay initiating ART		31 (23)	16 (2)	34 (10)	35 (11)
Ever relapsed		22 (16)	31 (4)	24 (7)	16 (5)
Adherence problems experienced (past/present)					
Pill-related (memory, side effects)		49 (36)	69 (9)	52 (15)	39 (12)
Psychosocial (stigma, depression, stress)		30 (22)	46 (6)	31 (9)	23 (7)
Structural (insurance, housing, drugs, jail)		23 (17)	38 (5)	28 (8)	13 (4)
Any current adherence problems		52 (38)	85 (11)	55 (16)	35 (11)
Expressed intrinsic motivation		91 (67)	92 (12)	93 (27)	90 (28)
Motivated by FI to change behavior		23 (17)	23 (3)	38 (11)	10 (3)
Found FI to be a nice reward		27 (20)	8 (1)	48 (14)	16 (5)
Uses adherence aids		70 (51)	69 (9)	79 (23)	61 (19)

^a^Includes two current relapsers who moved from VS to 57 and 71% of tests being virally suppressed

Sub-study participants received between two and eleven VL tests over the duration of the HPTN 065 study (mean 6.6). Sub-study participants joined the FI intervention with varying lengths of experience on ART. For example, only 13 of 73 participants were ART-naïve prior to exposure to the intervention. Greater than half of sub-study participants described being diagnosed at least a decade ago, some during the mid-1980s to mid-1990s. Most of the participants were diagnosed as adults, while four reported that they were diagnosed as children. About a third of participants described more recent diagnosis, from 2007 onward. The timing of and circumstances around HIV diagnosis were unclear for several participants.

### Pre-contemplation

Because all participants had initiated ART prior to or as a part of the HPTN 065 study, none could be characterized as in the pre-contemplation stage at the time of enrollment in the sub-study. Nevertheless, during their interviews about a third of participants (n = 23) described periods of pre-contemplation, several months or longer after receiving their HIV diagnosis when they had not considered going on ART (Table 2). The majority were diagnosed early in the epidemic; many described treatment delays of 2 to more than 10 years. Delays, as portrayed in the quotes below, were often attributed to fear of taking medications that were perceived as toxic. In some cases, participants were also dealing with drug or alcohol addictions or debilitating illnesses that prevented them from seriously contemplating taking their medications. Two participants, asked about when they first started taking HIV medications, described the following:

I was diagnosed in 1990. I did not start taking medication for about 10 years. So, around 2000 I started taking medication. And my concern was … I was concerned about taking any medication. Well, back in the 90’s, I’ve had friends who have gone through so much hell taking medication, and I felt that, as long as I wasn’t sick … as long as I wasn’t sick, I was going to just prolong it as long as I could. (66-year-old gay Black man)

Whew, 1992…. That’s when I found out I was positive…. That’s when that … what medicine was that that was out back then? What was the name of that medicine? They was saying it was kind of toxic…. And at that time I was getting high, you know, from crack, so I wasn’t really taking my medicine. You know what I’m saying. And I would end up in and out of the hospital a lot, because I was … you know, I was getting high off of crack, and then I was getting high. I’d get my check, I would get high, then I’d get sick and go in the hospital. And then, it seemed like every time I went in the hospital, my money would come. (54-year-old straight Black woman)

Despite important advances in HIV treatment, a number of participants diagnosed more recently, since 2007, still reported delays, although these were usually of shorter duration—from 6 months to 2 years. Recently diagnosed patients were less likely to attribute their lack of treatment to the drug regimen directly, but described needing some time before they could think about taking the medication, mostly due to shame, denial or depression. A young Hispanic man who was diagnosed around age 21, but who began treatment several years later described his emotional outlook at the time of his diagnosis:

Cause the way I found out was an awkward situation. It was right after my 21st birthday – like literally right after. It was two guys that I was messing around with at the time and usually I was like never really like that. […] And it just seemed like the one slip or the one particular time kind of like screwed me over for life…. I felt like the sky was falling. I felt like the walls were caving in. It was a very dark period for me. (26-year-old gay Hispanic man)

Similarly, a 30-year-old mother of two who began ART within the context of the study explained:

When I found out I had to start taking medicine it was a big shocker for me because, it was like… I wasn’t in denial about my illness but suddenly everything became real, because knowing you have an illness and not feeling certain symptoms and now taking the pills is like a reminder every, every day. So it was very hard for me. I went back and forth with my doctor, letting her know I was having some trouble with, with coming to terms in taking the medicine. So, she actually prescribed it and I didn’t take it for about two months and a half… So I told my doctor like you know maybe they should start like a program to prepare patients who don’t take medicine right away because I was very depressed. (30-year-old straight woman whose race was not reported)

### Low Adherence Group

Of 13 participants classified in the Low Adherence Group, two-thirds were women and four had been diagnosed with HIV as infants or young children, and just over half (n = 7) had been on medication for a decade or more.

All participants in the Low Adherence Group described their own adherence as mixed or low. They admitted to forgetting pills and some described being willfully non-adherent at times. For example, when asked how long she had been taking her HIV medication, a young transgender person responded:

I had them ever since I was 18 because that’s when I found out. But I took them a couple of times, then they started making me feel sick. I didn’t like that. So I recently started taking… so I’m like this person, I’m off and on. So I’ll start it and then stop it. […] So last year I got Complera, the one pill, and it is amazing. So I take it every night. I was undetectable in December, and for… December, January, and February, I was taking it. I stopped in the middle of February, and I started back last week… last Monday. So that’s how I am, but I’m … I’m continuing to be on it. (23-year-old straight Hispanic transgender woman)

Many in this group attributed their difficulties with adherence to depression or sadness often related in some way to relationship issues. Some experienced on-going challenges with drug, alcohol use or sexual risk behaviors. In addition to their current struggles with adherence, four of the nine participants diagnosed as adults described one or more periods of relapse in the past. For example, a middle-aged single Black woman, who acquired HIV from her partner of 4 years, continued to face setbacks when she remembered how she became infected. She said:

And I try to take care of myself [the] best way I know how. Sometimes I do forget my medication because I’m so busy all over the place, but when I do take it I make sure I take it. You know, I try to take it every day. I try not to miss a day. But sometimes when you’re very, very busy – cause at one time I just sat home, I would not take the medication, I’m a tell you that. I don’t know if I was, I don’t know what my mental state was at that time and I really believe that I just didn’t care. Because I just couldn’t come to grips of why that man would do that to me. (48-year-old straight Black woman)

The words of a 57-year-old mixed-race Hispanic man conveyed a continuing sense of guilt and stress that appears to affect his ability to adhere to his medication.

Interviewer: Really, why didn’t you want to take them?

*Participant: Tough with me, ‘cause I did it to myself. Ain’t nobody pushed me, I wish I would have known, I wish I would have known before. Doing all that stupid shit.*

Interviewer: And you were taking them before?

*Participant: Right, so the only thing is, I was like skipping one day, taking them a week, and skipping a Sunday…*

Interviewer: So, how do you think the gift card program changed that?

*Participant: Well, when I knew about the gift card, I was on top. I had stopped taking medication. It wasn’t because of the gift card. It was because things were going on and, and it’s like stress. (I’m) Really aggravated at myself sometimes. I’m always by myself.* (57-year-old straight Hispanic man)

Only one Low Adherer linked the potential to earn gift cards as an incentive to be more adherent. Most participants in the Low Adherence Group (n = 9) did not identify any role of the FI in incentivizing adherence. Several (n = 3) suggested that while the FI didn’t change their adherence much, it was a nice thing to receive some extra cash.

Well, I was taking it regularly and I mean, you know, I guess the impact that it had was you know like an extra seventy bucks or whatever the case was. But I mean, you know, it wasn’t something where I was taking the medicine because of the gift card. I mean, you know, I was working and stuff like that so money wasn’t really an issue. I guess it was just like, you know, an extra seventy bucks so why not do it? (26-year-old gay Hispanic man)

Although the misuse of the gift cards was reported uncommonly, one participant admitted that he had tried to use the card to support his drug habit. He explained:

I was acting up crazy, I was losing… you know what I’m saying? I was trying to figure how I could get over and get another card, and sell it, and get high again, you know what I’m saying? So I go, they shut me right down. I was glad. (58-year-old straight Black man)

Participants in the Low Adherence Group reported that, more important than the FI, the support of family and friends—and sometimes the admonitions of providers and others—reminded them to think about their medications. As one woman, who attributed her struggles with adherence to deep depression, described:

I was talking to my doctor and she was so mad with me. She said, ‘Sometimes it goes up and sometimes it goes down. You ain’t taking your medication.’ I said, ‘For real I don’t like taking the medication, I really don’t.’ Then she said, ‘Please just try to take this medication.’ (47-year-old straight Black woman)

Although “rough” on her, she appreciated the consistent care provided by the clinic staff who used to “sit [her] down all the time” to explain how medication adherence related to VL and to help her strategize ways to become more adherent.

### Action Group

About two-fifths of sub-study participants (n = 29) were either ART-naïve (n = 4) or not virally suppressed (n = 25) at baseline, but had VS in the majority or all VL tests conducted during their follow-up visits after the FI were initiated. Almost all assessed themselves as being good or even excellent adherers currently, but acknowledged that adherence could still be a challenge. Unlike those in the Low Adherence Group, these participants reported that small lapses strengthened their resolve. As one participant explained:

Well, I want to sustain it [my low viral load]. That’s why I continue to take it without having any problems. There are some times when I miss a dosage, especially at night. I feel bad about that. But I just do the next day and … after I come to the clinic and they do the blood and they find out that I’m still non-detectable, [it] makes me feel good…. I may fall asleep before it’s time. Then I wake up in the middle of the night and say, well I’ll just start again the next day. (58-year-old gay Black man)

Similarly, a 54-year-old gay Black man explained:

Now, it’s like clockwork. In the morning, I’ll take my medicine. In the evening, I’ll take my medicine. Two times I had the medication. Two times.”

Half of participants in the Action Group had been diagnosed 10 or more years before. Many faced significant barriers to ART use in the past; HIV-related fear and stigma prevented some from disclosing their illness or seeking treatment, and others found the number of pills and severity of side effects to be daunting. For some, having navigated these earlier challenges made it easier to adhere to their current medications. A 48-year-old gay White man reflected on those earlier days:

My experience with them has changed, because they’ve gotten more tolerable and more … easier … easier to take, fewer pills and many fewer side effects that are visible… Side effects from pills when they’re bad were horrible.

Others in the Action Group had been diagnosed more recently. Several ART-naïve patients suggested that transitioning to ART was more difficult for them than the diagnosis itself.

I felt like it was annoying. It was like I went from taking no pills to taking like this one pill and it was like, I don’t know. I didn’t like taking it because I had to make sure I took it every day. Then like I had like these really dumb side effects like really different….Because it’s a big adjustment to take pills every day when you are not use to taking any pills because like sometimes you forget. […] Like sometimes, I take them in the morning so like if I forget until like night, it’s almost morning again. So I be like, if I take one now and one in the morning, I feel like I’m overdosing. (18-year-old gay Black man)

More than a third of the Action Group (n = 12) reported that they were motivated to be or to remain “undetectable”. Having “undetectable” VL was both a sign of health and an indication of good behavior, even when some participants did not fully comprehend how being “undetectable” was determined.

Because I know I’m not going to be 100% but when I get to undetectable, where I get the benefit, that’s priceless. So I said you know what? To whom much is given much is required. Take a little bit of extra time, go back and do that. (54-year-old gay Black man)

I’m undetectable. And that’s the viral load…. I didn’t want to know too much about it, but then as I, as I started taking the (medicine), and I was like I want to know more about it. So, that makes me feel good that I’m on it. (38-year-old bisexual Black woman)

The majority described strong intrinsic motivations to take their medications. They wanted to “stay healthy” or “to live” either for themselves or, for some, for their own children or grandchildren. One difference between those who did and did not achieve 100% VS over time appeared to be the degree to which they accepted ART as a part of their lives. Most suggested that “it is no big deal”. One patient considered himself “blessed” to be able to take the medication.

The majority of participants in the Action Group had adopted explicit strategies to facilitate better adherence. For example, almost half of the group described making specific changes to their routines to support their adherence. This included changing the timing or the way they took their medications, setting alarms or using pill boxes.

I take it like aspirin. Because I have one of those 7-day pill boxes. It’s a big one. I got it from [the nurse] here at the clinic. It has AM and PM and see I take anti-depressants. I also take another, I take something for prostate, I just can’t remember all the stuff and another antibiotic for HIV-positive people so you don’t get like thrush, things like that. I just take them like I was taking… I just take them every day like I’m supposed to, that’s it. First thing when I get up in the morning, I have a little something to eat, I take my morning pills and at night, usually after I have dinner, I take my evening pills. I do that every, single day. I don’t even think anything about it. It’s just what I have to do and I do it. (60-year-old bisexual White man)

A young married, Black woman reflected on the changes she had made to become more adherent, saying:

Well when I first started taking it, honestly, I didn’t take it serious like how I do now. Yeah, I didn’t. I have an alarm now that I set to take it so I don’t miss. Back then it was like I tried to put it out my head, because, you know, being diagnosed with that is not easy. (22-year-old straight Black woman)

Finally, most participants (n = 25) in the Action Group found FIs to be motivating to achieve adherence. Most were clear about the importance of taking their medications as they were prescribed. But, many also indicated that remaining adherent was hard, and FIs made it a little easier. They described it as an incentive, a reward or an extra nudge. For 11 participants, FIs had an even stronger influence on adherence. For example, a 50-year-old straight Black man explained:

I think the card program too, even though you’re sick, it still helps you to push yourself to do it, to take your meds to try to stay undetected with you, with the card or without the card. But after a while with the card, you realize you could do it if you getting the card or not. But the card helps start that force of you pushing yourself.

### Maintenance Group

Two-fifths of the participants in the sub-study were already on ART and virally suppressed at the time they entered the HPTN 065 study (n = 31). All but one participant in this group described a high level of commitment to being adherent to their medication currently—even when it meant overcoming barriers. They made declarations like “It doesn’t control me”; “I still keep taking my medicine no matter what”, and “I know what I got to do.” Most appeared to have committed themselves to the process of adhering to treatment. They described filling their own pill boxes, arranging their schedules to accommodate pill-taking, and finding ways to take their pills even when away from home. For example, a participant asserted:

I, my decision to go ahead and start taking the medications was an informed decision. It wasn’t a decision out of panic, it wasn’t a decision because ‘the doctor said so’, I was informed because I did my research. I felt like, this, they’re gonna give me one pill, one pill a day. I can do that, I already know about the Atripla, what’s in it, I can live with that. Let’s go ahead and do it, so it was an informed decision. (49-year-old gay Black man)

Others described similarly high levels of commitment.

I know that I need to take it first thing in the morning. I know I can’t take it on an empty stomach. You know, I have to eat breakfast, so it’s easy for me to develop a regimen for myself. So it becomes natural. (66-year-old gay Black man)

I always carry my pills in my pocket. …Well, you know what? I’m … I’m very … very picky when it comes to stuff…. I have this thing of taking it 12 midnight, 12 afternoon, 12 midnight, 12 afternoon. So I know that when 12 o’clock hits, or 12 afternoon like today, I have in my pocket, get a glass of water, drank it, take my pills, I’m good. So I already have this thing of carrying them on me if I’m going out. (33-year-old gay Hispanic man)

At least half of patients used words like “habit” or “routine”—equating adherence to ART with “taking a daily vitamin”. In his exchange with the interviewer, a 38-year-old gay Black man describes his no-excuses approach:

And, like I said, it’s become habitual, so I don’t even, you know …Think about it? When that time sets, it’s like, okay, get this done and move on, you know? […] So, I, listen, wherever I’m at, if it’s time, it’s time. It’s going down, right there.

This combination of commitment and habit is apparent in the words of a 55-year-old gay White man as well:

It’s like shaving… you have to do it every single day, but sometimes you just don’t feel like dragging a metal blade across your face because you know you’re going to have… it’s going to be raw, and it’s going to hurt your face. Same as HIV medications, you know you have to take them, but, you know, is this the day that the HIV medications upset my stomach, or is it not? I mean even after 25 plus years of taking the medications, I occasionally have side effects, so you know… so that’s how I equate it… Routine… knowing that I wake up in the morning, I eat something. I take my medications.

Almost all participants in the Maintenance Group described using at least one, but often multiple, adherence aids to support their adherence behavior. Most common was establishing a routine around taking their medications (n− = 12). Additionally, a number of participants (n = 8) specifically mentioned using pill boxes, phone alarms or other techniques.

To a greater extent than in the other groups, participants in the Maintenance Group tended to reflect on the barriers they had overcome—the positive changes in their medication regimens and consequently the positive feelings they had about their regimens. Many also identified how the love and support of others around them—from family members, sexual partners and healthcare providers—helped keep them focused on being adherent. A 51-year-old man reflected on the acceptance and support his family extended to him, when he returned home one holiday after having contracted HIV.

Well, they … they didn’t know for years that I was HIV (positive) until I got sick, you know. Then my sister, you know, she [noticed] the way I looked, and I didn’t think she would talk to me. You know, I didn’t want to come, you know. So, I came to visit for the holiday, Christmas. They didn’t want me to go back. So, we went back, packed up my stuff, you know. She made me, you know, ‘Here’s your medicine.’ Every day, and, you know, ‘Take care of it.’ you know. And they all knew – all my family knew…. The fact that now, there’s more people I know who are on meds. You know, it’s like I’m not alone doing this. … It’s like a support group, you know, that helps, you know. (51-year-old gay Hispanic man)

Several women in this group talked about the important role their husbands or partners played in reminding them about taking their medication. For example, a 56-year-old married woman explained:

My husband’s like ‘Did you take your medicine today?’ ‘Yes I took my medicine.’ He’s such a wonderful man. ‘Did you take your medicine?’ And I say ‘I took my medicine.’ He gets checked. We don’t, I mean we’re older now you know what I’m saying but- and I tell him every three months he gets checked. (56-year-old straight woman whose race was not reported)

None of the participants in the Maintenance Group expressed negative attitudes towards FIs. The majority felt that it was good to provide FI, especially for those who had a problem adhering. They thought it was “a good idea for kids, especially kids who […] don’t learn to take their medicine” or for those who “deviate away from the drug use, or lack of self-preservation and self-esteem.” However, only a small number (n = 3) felt that FIs helped them improve their adherence. They tended to describe the program as ‘a nice incentive’ for something they were already doing. About half of the group (n = 16) were clear that their intrinsic motivation and having integrated medications into their daily routine—and not the FI—drove their own adherence.

No. I told you I would’ve done it without the gift cards…. I just know to take my medicine that’s all. I’ve been going through this for many years now, I should be used to the grind, you know? (56-year-old straight Black man)

The gift card, you know, didn’t drive me, you know. I mean it helped me, but I was driven because I wanted to live longer, you know. I mean, that’s why I come to see the doctor and that. (56-year-old straight Hispanic man)

Personally, for me, the gift card was just another … kind of the icing on the cake. I’m the kind of person that I’m … I’m concerned about my health. I’m going to take care of me…. [That] didn’t change, because I know that I have no choice, and I love myself, and I’m going to do what I need to do for myself. (66-year-old gay Black man)

A few in the Maintenance Group (n = 5) expressed some reservation about FIs. For example, one participant in this group worried that ‘Not all people do the right thing with the money that they do get. You know, they do drugs, so …’, while another worried that FIs might ‘create a division’ between those who can and cannot reduce their VL.

### Relapse

Only two participants had VL suppression at baseline and did not have sustained VS during study follow-up. In addition, 16 participants who were currently in the Low Adherence, Action or Maintenance Groups described relapse in adherence prior to joining the study. More than half (n = 10) had been diagnosed and began treatment prior to 2007—most, very early in the epidemic. The reasons for relapse included incarceration or homelessness, drug addiction (crack or crystal meth), excessive alcohol use, and lack of health insurance or money for day to day needs.

I was taking my medication when I was incarcerated. Then when I came home, I actually stopped. I was taking them here and there, but I was at a [place] where I didn’t have my own place. I was going from house to house, street to street, bench to bench […] I didn’t want people I was living with to find out, so I end up doing the rest of my jail time for violating probation, so I end up going back to jail. I didn’t take no medication at the time ‘cause I was just too embarrassed at the time to take my medication […] And I think my last bit when I did, I came home and that’s when I actually started volunteering for Whitman Walker, truthfully. So they had a program at the time where they was holding my medication. I come here and take my medications. […] So while I was volunteering to come here doing that, that’s when my CD4 count starts to actually going up but then it went down again ‘cause I didn’t (get) incarcerated but I just started being depressed because I was still (going) from house to house, program to program at the time. (31-year-old gay man whose race was not reported)

About half of participants who reported previous relapse in adherence (n = 9) attributed it to a willful decision to stop taking the medications—either because of side effects or because the individual couldn’t see how the medications were helping. Reflecting back to those periods of time, several described themselves as “becoming my own doctor.” Often, a decline in health status led them to begin taking their medications again.

Finally, a few participants admitted that the end of the gift card program itself led to a relapse. For example, a 22-year-old straight Black woman stated:

Yeah, it (the gift card program) was effective for me, because, you know, it does bring you up a little bit, you know. But it stopping… actually, when it stopped, I stopped my medication. I’m going to be honest. I stopped for a couple of months because I was having… it felt like that stopped, and then I had insurance problems.

## Discussion

Our sub-study provided an in-depth understanding of the patterns of medication adherence experienced by patients participating in an FI intervention, including their perspectives on the role that FIs played in promoting or sustaining their adherence. Over a third of sub-study participants were already on ART, were VS upon entering and maintained VS throughout the FI intervention. Similarly, three-fourths of those who were ART naïve at baseline maintained 100% VS throughout the study. Although generally appreciated, the FIs appeared to have little impact on the adherence-related motivations and behaviors of this group. For others who were on treatment but unsuppressed at baseline, or were ART-naïve but unable to maintain VS throughout the study, FIs provided the additional “nudge” needed for many to better integrate behaviors that would help them become and remain “undetectable”. However, the FIs were insufficient to help a small group of Low Adherers to overcome adherence barriers.

Participants described a range of barriers to medication adherence, including those related to the medication regimen itself, as well as psychological, social and structural barriers. Across the Maintenance, Action and Low Adherer groups, participants differed in how they confronted barriers and made use of facilitators. They also differed in how they viewed the FI program and its effect on their adherence.

Fear of health-related side effects played a strong role in delaying (pre-contemplation) or temporarily stopping (relapse) treatment. While pill regimens have become much simpler, participants across the three sub-study stages described some difficulties with daily pill-taking due at times to the specific regimens. Unlike those in the Low Adherence Group, many participants in the Action Group actively worked to overcome their barriers by adjusting the timing or the way they took their medication and, if missing an occasional pill or two, recommitting themselves to pill taking. Maintainers appeared to have fully routinized their pill-taking regimens and rarely let life circumstances get in the way of pill-taking routines. These findings are consistent with other studies that attributed early discontinuation of ART regimens to intolerance or toxicity concerns [28], or found that characteristics of the medication regimen including dosing frequency, pill burden and type of ART were correlated with medication adherence [5, 29].

Psychosocial factors appeared to play a bigger role than medication-specific concerns in non-adherence. For those who were in the Low Adherence Group, pill-taking was often a reminder of their HIV status and how they got HIV. Such reminders led some in this group to miss pills or to stop taking their pills altogether. In a recent meta-analysis of potential predictors and correlates of ART adherence concluded that psychological factors were a stronger predictor of adherence than dosing regimen [5]. Psychosocial factors including HIV-related stigma, anxiety, depression and stress have been associated with low adherence to medication [30–33], including among adolescents [31], HIV sero-discordant couples [34] and women and minorities [35]. In our study, it is interesting to note that the Low Adherence Group consisted of a higher proportion of women and four of seven participants who were diagnosed as children.

Several authors have argued that the field of HIV treatment has not paid sufficient attention to structural issues—either from theoretical or empirical perspectives [36–39]. Indeed, in our study structural barriers, including lack of employment, drug and alcohol addiction, incarceration and periods of homelessness also emerged as important reasons for non-adherence and relapse. Suggestive of these barriers, sub-study participants in the Low Adherence Group had lower levels of education and income; several described ongoing struggles with alcohol or drug addiction. Incarceration, periods of homelessness and lack of health insurance were also experienced by participants in the Action and Maintenance Groups, although most often when describing initial delays in treatment or periods of non-use in the past. Having traversed such difficult periods became a source of power for some.

In this study, FIs were delivered within clinic settings that offered additional supports, including information about VL, strategies to routinize pill-taking behavior, and medical services to address mental and physical health issues. The role of adherence facilitators, including FIs, varied across the SOC continuum. Participants in all three groups (Maintenance, Action and Low Adherer) described making changes to their daily routines and use of adherence aides, including pill boxes and alarms. However, those in the Action Group were more likely to describe an active role—in filling their own pillboxes or making adjustments to their pill-taking routines—than participants in the other two groups. Similarly, the Action Group drew a more explicit connection between receiving FIs and double-checking whether they had taken their pills. In contrast, the FI was not seen as necessary to those in the Maintenance Group to incentivize a behavior that they already had squarely under their control. Among the Low Adherence Group, support from clinic providers and others to educate, strategize and overcome other health challenges appeared critical. The amount of the FI offered through the HPTN 065 study was not considered sufficient by some in the Low Adherence Group to overcome the significant barriers to adherence they faced. This finding was similar to a 2013 feasibility study, in which VS increased for those with previous detectable VLs from 57 to 69%, but there was no impact on those who had never achieved suppression prior to the intervention, which suggests that the amount of the incentive was not adequate to merit behavior change [40]. Our sub-study findings also align with the conclusions of the HPTN 065 main study, which found that FIs increased VS by approximately 4% at sites that offered them, but higher (5%) among patients not consistently VS prior to study enrollment [25].

This sub-study has several limitations. First, an individual’s stage of change is typically assessed using a standardized questionnaire that evaluates his/her readiness to change [12]. Because our sub-study did not administer a structured SOC tool to assess participants’ attitudes towards ART medication adherence, we used an outcome measure—the percent of VL tests identified as virally suppressed—applied retrospectively to categorize our study population into SOC-like stages. Nevertheless, the categorization allowed us to examine and compare the self-reported influence that individual, social and structural factors, including offering an FI, had on achieving and maintaining VS. Second, almost half of our sample had long-term experience taking ART and entered the study with a history of VS. Consequently, the role of FIs may be less important to this group. Furthermore, their recall of adherence-related barriers may have decreased with time. Nevertheless, the group of long-term ART users does provide a useful contrast by which to examine the attitudes and behaviors of those who were unsuppressed or new to ART at the beginning of the study. Finally, taken as a whole, this qualitative sub-study provides an in-depth understanding of adherence-related barriers faced by patients at different stages of ART adherence, of the strategies they use to facilitate ART adherence and the role that FI play in adherence support.

## Conclusion

FI effectiveness may vary across the SOC continuum. FIs may have greatest impact for those who are ART naïve or have not yet formed explicit adherence routines or habits, such as those in our Action Group. FIs may be insufficient on their own to overcome adherence lapses when strong social or structural barriers are present however, their use as part of comprehensive care strategies that provide broader mental health, economic and social services could prove effective and should be further researched. Finally, FIs are probably unnecessary for those who have strong intrinsic motivations and well-integrated habits to support adherence.

## Footnotes

1 The local IRBs reviewing this study were: Albert Einstein College of Medicine of Yeshiva University IRB, Children’s National Health System IRB, and George Washington University and Medical Center IRB.

2 One interview was partially conducted in Spanish.
